# Cardiovascular magnetic resonance for the detection of descending thoracic aorta calcification in patients with end-stage renal disease

**DOI:** 10.1186/s12968-021-00769-6

**Published:** 2021-06-24

**Authors:** Elbert Edy, Alastair J. Rankin, Jennifer S. Lees, Pauline Hall Barrientos, Rosemary Woodward, Sokratis Stoumpos, Ioannis Koktzoglou, Robert R. Edelman, Aleksandra Radjenovic, Patrick B. Mark, Giles H. Roditi

**Affiliations:** 1 Institute of Cardiovascular and Medical Sciences, BHF Glasgow Cardiovascular Research Centre, University of Glasgow, Glasgow, G12 8TA UK; 2 Renal and Transplant Unit, Queen Elizabeth University Hospital, Glasgow, UK; 3 Department of Clinical Physics and Bioengineering, NHS Greater Glasgow and Clyde, Glasgow, UK; 4 Clinical Research Imaging, NHS Greater Glasgow and Clyde, Glasgow, UK; 5 Radiology, NorthShore University HealthSystem, Evanston, IL 60201 USA; 6 Radiology, University of Chicago Pritzker School of Medicine, Chicago, IL USA; 7 Radiology, Feinberg School of Medicine, Northwestern University, Chicago, IL USA; 8 Department of Radiology, NHS Greater Glasgow and Clyde, Glasgow, UK

**Keywords:** Thoracic aortic calcification, End-stage renal disease, Cardiovascular disease, Computed tomography, Magnetic resonance imaging, Radial volumetric interpolated breath-hold examination (radial-VIBE) sequence

## Abstract

**Background:**

Vascular calcification is an independent predictor of cardiovascular disease in patients with chronic kidney disease. Computed tomography (CT) is the gold-standard for detecting vascular calcification. Radial volumetric-interpolated breath-hold examination (radial-VIBE), a free-breathing gradient-echo cardiovascular magnetic resonance (CMR) sequence, has advantages over CT as it is ionising radiation-free. However, its capability in detecting thoracic aortic calcification (TAC) has not been investigated. This study aims to compare radial-VIBE to CT for the detection of TAC in the descending aorta of patients with end-stage renal disease (ESRD) using semi-automated methods, and to investigate the association between TAC and coronary artery calcification (CAC).

**Methods:**

Paired cardiac CT and radial-VIBE CMR scans from ESRD patients participating in 2 prospective studies were obtained. Calcification volume was quantified using semi-automated methods in a 9 cm segment of the thoracic aorta. Correlation and agreement between TAC volume measured on CMR and CT were assessed with Spearman’s correlation coefficient (ρ), linear regression, Bland–Altman plots and intraclass correlation coefficient (ICC). Association between CAC Agatston score and TAC volume determined by CT and CMR was measured with Spearman’s correlation coefficient.

**Results:**

Scans from 96 participants were analysed. Positive correlation was found between CMR and CT calcification volume [ρ = 0.61, 95% confidence interval (CI) 0.45–0.73]. ICC for consistency was 0.537 (95% CI 0.378–0.665). Bland–Altman plot revealed that compared to CT, CMR volumes were systematically higher at low calcification volume, and lower at high calcification volume. CT did not detect calcification in 41.7% of participants, while radial-VIBE CMR detected signal which the semi-quantitative algorithm reported as calcification in all of those individuals. Instances of suboptimal radial-VIBE CMR image quality were deemed to be the major contributors to the discrepancy. Correlations between CAC Agatston score and TAC volume measured by CT and CMR were ρ = 0.404 (95% CI 0.214–0.565) and ρ = 0.211 (95% CI 0.008–0.396), respectively.

**Conclusion:**

Radial-VIBE CMR can detect TAC with strong positive association to CT, albeit with the presence of proportional bias. Quantification of vascular calcification by radial-VIBE remains a promising area for future research, but improvements in image quality are necessary.

**Supplementary Information:**

The online version contains supplementary material available at 10.1186/s12968-021-00769-6.

## Background

Patients with chronic kidney disease (CKD) have a greater risk of cardiovascular disease (CVD) and all-cause mortality compared to age-matched controls within the general population [1]. This risk is greatest in patients with end-stage renal disease (ESRD) who require renal replacement therapy in the form of dialysis or a kidney transplant [2]. Traditional cardiovascular risk factors (e.g., diabetes, hypertension, hyperlipidaemia), are prevalent amongst CKD populations. In addition, there are CKD-specific risk factors that contribute to the predisposition to CVD, such as excessive arterial calcification and vascular stiffening. In ESRD patients, aortic stiffness is higher compared to healthy controls [3], and arterial calcification may be partly responsible [4]. Clinically, both arterial stiffness and calcifications have been shown to be independent predictors of CVD mortality in ESRD patients [5, 6] and have been used as a surrogate end point for clinical trials in this population [7].

The thoracic aorta can be imaged in both cardiac computed tomography (CT) and cardiovascular magnetic resonance (CMR) and quantifying thoracic aortic calcification (TAC) may have some utility in improving risk prediction. In primary prevention cohort studies, TAC was shown to be an independent predictor for all-cause mortality [8, 9], but not for CVD events [10–13]. While in patients with stable angina, TAC was associated with increased risk of death and CVD [14]. Although data in ESRD patients are lacking, other arterial calcifications, such as coronary artery calcification (CAC) and abdominal aortic calcifications, are associated with higher risk of all-cause and cardiovascular mortality in advanced CKD (stages 4–5) and haemodialysis patients [15, 16]. Therefore, it is plausible that TAC might also be a risk factor for CVD events in ESRD patients.

CT is a well-established imaging modality for detecting vascular calcification in clinical practice. Calcification can be quantified on CT using the Agatston score [17], which takes into account the area and density of calcified lesions, or the volume score, which does not depend on calcium density [18]. Traditionally, calcification is hard to discern with conventional spin-echo sequence in CMR because it appears with various signal intensities [19]. Moreover, calcification is hypointense due to low proton density and often lies adjacent to the dark arterial lumen [20].

Recent work has shown that a prototype proton density-weighted in-phase stack-of-stars CMR using a small flip angle gradient-echo readout can accurately quantify aorto-iliac and ilio-femoral vascular calcifications [21, 22]. A similarly configured and commercially-available gradient-echo CMR sequence called radial volumetric interpolated breath-hold examination (radial-VIBE) could therefore serve as a potential alternative to CT in detecting and quantifying vascular calcification without ionising radiation [23]. However, whether this could be applied to detecting TAC is unknown.

The aim of this study was to compare radial-VIBE to CT for the detection and quantification of TAC, specifically in the descending thoracic aorta, in patients with ESRD using a semi-automated approach. In addition, the association between descending TAC and CAC was investigated.

## Methods

### Sources of images and other clinical data

Imaging data from adult patients with ESRD participating in 2 prospective research studies was used: (1) Vitamin K in kidney transplant organ recipients: Investigating vEssel Stiffness (ViKTORIES) trial (Current Controlled Trials number, ISRCTN22012044) and (2) The Interrogation of the Cardiomyopathy of Chronic Kidney Disease With advancEd caRdiac Imaging (TICKER) study (ClinicalTrials.gov number, NCT03704701). All subjects gave written informed consent to the respective studies, which were reviewed and approved by the local Research Ethics Committee. The ViKTORIES trial [24] is a phase II, double-blinded, parallel-group, randomised, placebo-controlled trial comparing vitamin K supplementation to placebo on vascular stiffness in renal transplant patients. The TICKER study is an ongoing observational study that is assessing the effects of dialysis on the myocardium using CMR. Both studies collected imaging data, of which the paired cardiac CT and CMR (acquired within 24 h of each other) were used in the current study. For both VIKTORIES and TICKER studies, the assessment of calcification was one of several prospectively defined research questions being addressed. The paired scans were performed at the Clinical Research Imaging Facility based at the Queen Elizabeth University Hospital in Glasgow.

### CT image acquisition

Electrocardiogram (ECG) gated non-contrast scans of the heart were acquired at 120 kVp in a single heartbeat scan using an Aquilion ONE Vision Edition CT scanner (Canon Medical Systems Ltd., Crawley, UK). Radiation dose was reduced by using the Adaptive Iterative Dose Reduction 3D reconstruction algorithm.

### CMR image acquisition

Non-contrast CMR images were obtained using a 3 T scanner (Prisma, Siemens Healthineers, Erlangen, Germany) with an 18-channel surface coil placed anteriorly, and a 32-channel spine coil placed posteriorly. Radial-VIBE images were acquired in coronal plane using the StarVIBE product (Siemens Healthineers), which is a free-breathing, 3D, proton density-weighted, stack-of-stars, gradient echo sequence. The imaging parameters used were: field-of-view (FOV) 462 × 462 mm, slice thickness 3 mm, repetition time (TR) 4.18 ms, echo time (TE) 2.46 ms, flip angle 2.5 degrees, acquired voxel size 1.2 × 1.2 × 1.2 mm, sampling bandwidth 720 Hz/pixel, scan time 4.30 min. No cardiac or respiratory gating were utilised.

### Quantitative image analysis

**Fig. 1:**
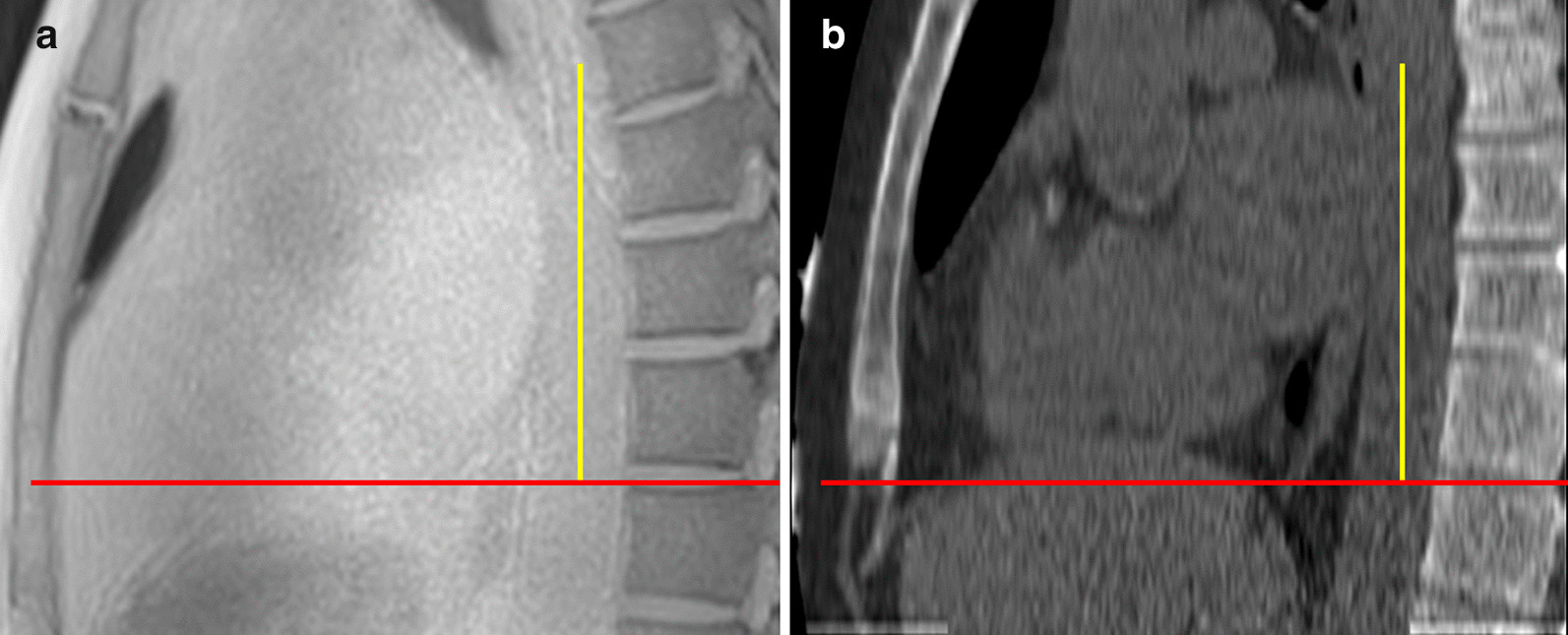
Sagittal (**a**) radial-volumetric interpolated breath-hold examination (VIBE) cardiovascular magnetic resonance (CMR) and (**b**) computed-tomography (CT) images of descending thoracic aorta. A 9 cm segment of thoracic aorta from the same patient is chosen on both CT and radial-VIBE images. Red horizontal lines mark the level of the top of the vertebra that is closest to the inferior surface of the heart. Yellow vertical lines correspond to 9 cm of thoracic aorta

One investigator analysed all of the images to measure calcification volume. A second investigator, who was added post-hoc, re-analysed a random sample of scans representing 10% of the cohort to assess the inter-observer reproducibility of our quantitative analysis protocols. To promote blinding, randomly ordered CT images were batch analysed before radial-VIBE images. The latter were analysed in a random order over a week later, without reference to CT results. Horos is a free and open source code software (FOSS) program that is distributed free of charge under the Lesser General Public License at Horosproject.org and sponsored by Nimble Co LLC d/b/a Purview in Annapolis, Maryland, USA. On sagittal views on both CT and radial-VIBE CMR images, Horos was used to select a 9 cm segment of the descending thoracic aorta, starting from the level of the top of the vertebra closest to the diaphragmatic surface of the heart and then progressing superiorly as shown in Fig. 1. If the vertebra was out of the FOV, then the area of analysis would begin from the inferior surface of the heart. The descending thoracic aorta was chosen as the region of thoracic aorta most reliably visualised on CT within a field of view allowing simultaneous imaging of CAC. The CT and radial-VIBE CMR images containing only the selected portion of aorta were exported and ImageJ (version 1.52q, National Institutes of Health, Bethesda, Maryland, USA) [25] was then used to detect and quantify the volume of calcification present within the descending thoracic aorta. Volume of calcification is the product of the area of the lesions detected and the slice thickness:

$${\text{Volume}}_{{\left( {in \, mm^{3} } \right)}} = \sum \left( {Area\, in\, mm^{2} *slice \,thickness \,in \,mm} \right)$$

### CT image analysis

**Fig. 2:**
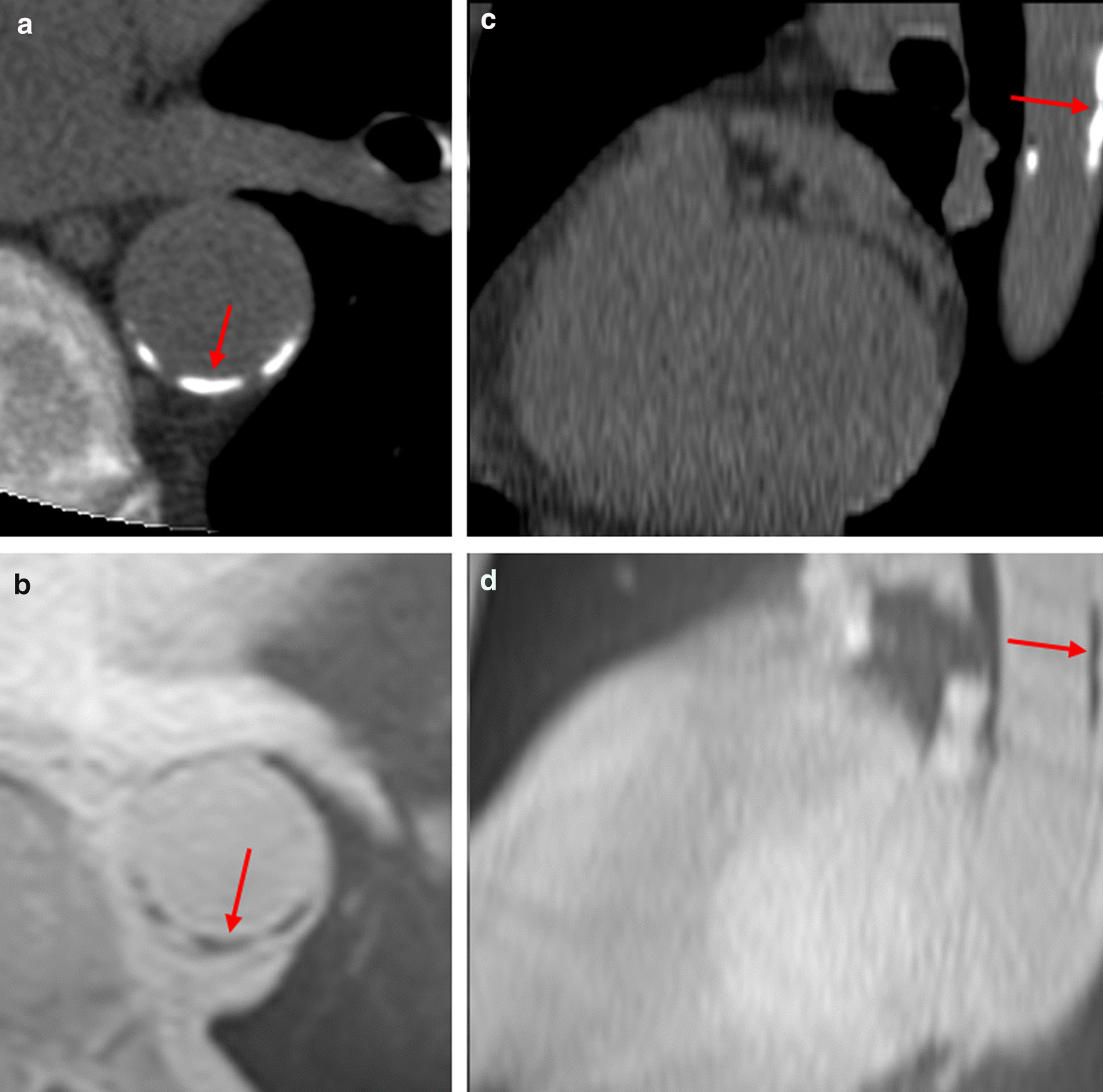
Representative images of calcifications on CT (**a**, **c**) and radial-VIBE (**b**, **d**). Images **a** and **b** are axial slices; Images **c** and **d** are sagittal slices. Calcifications are indicated by the red arrows. For this participant, volume of calcification detected by CT = 835 mm^3^, radial-VIBE volume = 634 mm^3^

CT images were reconstructed with 3 mm slice thickness and 3 mm slice interval to be used for analysis. Calcification was defined as voxels with attenuation values of 130 Hounsfield units (HU) or greater and appearing bright on CT (Fig. 2), this corresponds to approximately 2 standard deviations (SD) higher than the attenuation of unenhanced blood. Although arbitrary, this is accepted as the conventional threshold for CT assessment of arterial calcification [17]. A median filter of 3 mm radius was used to reduce ‘salt and pepper’ noise while preserving sharp edges. Once a threshold had been set to segment calcifications, a region-of-interest (ROI) was manually drawn around the wall of the descending thoracic aorta if calcified lesion(s) was present. Lastly, the area of calcification within the aorta was measured using automated thresholding. The patient’s total volume of calcification was then calculated offline. CAC Agatston scores were reported by a consultant radiologist in line with clinically approved protocols, blinded to treatment allocations and clinical variables using dedicated analysis software (Vitrea Advanced, Vital Images, Minnetonka, Minnesota, USA).

### Radial-VIBE image analysis

Reconstructed radial-VIBE images with 3 mm slice thickness and 3 mm slice interval were used for analysis. On the transverse plane, ROIs were manually delineated around the aortic wall in every consecutive slice. Next, a bespoke segmentation algorithm was used to segment calcifications (based on signal intensity) and measure their area. Aortic calcification appears hypointense (i.e., dark) on radial-VIBE images (Fig. 2) and was defined as a voxel with signal intensity of at least 2.5 SD *below* the mean signal intensity of voxels within the ROI (i.e., aorta) of each slice. A previous study*,* which utilised similar techniques, has defined calcification on MRI as between 2 and 3 SD below the mean signal intensity of the ROI [22]. In our cohort, 2.5 SD was chosen as it was quickly evident during the algorithm development that 2 SD insufficiently distinguished calcification from noise, while 3 SD excluded obvious calcification.

### Qualitative image analysis

Two observers (E.E. and A.R., with one and four years experience of researching vascular calcification, respectively) subjectively compared all (*n* = 96)radial-VIBE scans to CT. A 5-point Likert scale (1—very poor, 2—poor, 3—fair, 4—good, 5—excellent) was used to assess the confidence with which the radial-VIBE matched the CT with regards to calcification presence, location, size and shape [26]. Where scores disagreed, scans were reviewed to reach consensus.

### Statistical analysis

Data were analysed with Minitab^®^ Statistical Software (version 19.2.0.0, Minitab, State College, Pennsylvania, USA) and SPSS (Statistical Package for the Social Sciences, International Business Machines, Inc., Armonk, New York, USA). Normality of variables was assessed with P-P plot, Kolmogorov–Smirnov, and Shapiro–Wilk tests. Correlation between volume of calcifications in CT and radial-VIBE was assessed with Spearman’s rank correlation coefficient $(\rho )$, and linear regression. This is an exploratory, hypothesis-generating, study. The research question was prospectively defined and was considered in the design of the source studies. However, the sample size of the source studies was determined in relation to primary end points unrelated to the methods described in this report. A post-hoc power calculation was not performed.

Spearman’s rank correlation coefficient was also used to investigate the association between volume of aortic calcifications (detected by CT and radial-VIBE separately) and CAC Agatston score assessed by CT [27].

Bland–Altman plots were constructed, and mean bias (radial-VIBE volume minus CT volume) and 95% limits of agreement (LOA) were calculated between CT and radial-VIBE volume. However, as the assumptions of constant mean bias and standard deviation of differences were violated, linear regression was used to model the relationship between mean bias and the mean of calcium volume [28]. The standard deviation of the residuals from regression were then used to estimate 95% LOA.

To assess the degree of consistency and absolute agreement between CT and radial-VIBE volume, intra-class correlation coefficient (ICC) estimates and their 95% confidence interval (CI) based on a single-rating and 2-way mixed model were calculated. ICC was also used to measure intra- and inter-observer reliability of quantitative analysis by randomly sampling and reanalysing 10 paired CT and radial-VIBE scans after blinding of original volume scores. Weighted Cohen’s kappa (κ) coefficient was used to assess inter-observer reliability of qualitative scores.

### Sensitivity analyses

As a pre-specified sensitivity analysis, where there was a large discrepancy in the detected calcium volume between radial-VIBE and CT, the area of analysis was reassessed to check for any difference in ROI selection. A re-analysis of the volume of calcification would be done as part of sensitivity analysis if there was a clear discrepancy in area of analysis. This would remove blinding and therefore would be purely exploratory. Data points with standard residuals of 3 or more detected by linear regression were considered as outliers and a sensitivity analysis excluding them was performed.

## Results

### Baseline characteristics

A total of 96 participants was included in the analysis (24 from TICKER, 72 from ViKTORIES). Twelve were excluded from analysis (1 from TICKER, 11 from ViKTORIES) due to various reasons (See Additional File 1).

The relevant demographic and disease characteristics of the ViKTORIES and TICKER participants are shown in Table 1. The mean age of study participants was 59.6 years, a third were female and a third were current or ex-smokers (Table 1). Due to the selection criteria chosen for their respective studies, all the TICKER participants were receiving hospital-based intermittent haemodialysis, while ViKTORIES trial participants had a functioning kidney transplant. The median renal replacement therapy vintage was 2 years for TICKER participants and 7 years for ViKTORIES participants (Table 1). The mean estimated glomerular filtration rate (eGFR) for patients with a functioning kidney transplant (i.e., ViKTORIES participants) was 52.5 ml/min/1.73 m^2^ (SD: 21.8 ml/min/1.73 m^2^).

**Table 1:** Characteristics of TICKER and ViKTORIES participants at baseline

Characteristics	ViKTORIES N = 72	TICKER N = 24	Combined N = 96
Age, Mean ± SD* (year)	57.9 ± 8.9	64.7 ± 1.86	59.6 (9.3)
Male sex (%)	45 (62.8)	15 (62.5)	60 (66.7)
White race (%)	70 (97.2)	21 (87.5)	91 (94.8)
Diabetes (%)	18 (25)	11 (45.8)	29 (30.2)
Smoking status (%)			
Non-smoker	47 (65.3)	18 (75.0)	65 (67.7)
Ex-smoker	19 (26.4)	5 (20.8)	24 (7.3)
Current smoker	6 (8.3)	1 (4.2)	7 (25.0)
Previous cardiovascular disease^†^	17 (23.6)	11 (45.8)	28 (29.2)
eGFR, mean ± SD (ml/min/1.73 m^2^)^‡^	52.5 ± 21.8	–	–
Renal replacement therapy vintage (years)			
Median	7.10	1.96	–
Interquartile range	10.48	2.69	–

*SD denotes standard deviation

^†^Participants were considered to have previous cardiovascular disease if they had one or more of the following: history of ischaemic heart disease, heart failure, coronary revascularisation (including percutaneous coronary intervention and/or coronary artery bypass graft), stroke and/or transient ischaemic attack, and/or peripheral arterial disease

^‡^eGFR denotes estimated glomerular filtration rate

### Radial-VIBE vs CT volume

**Fig. 3:**
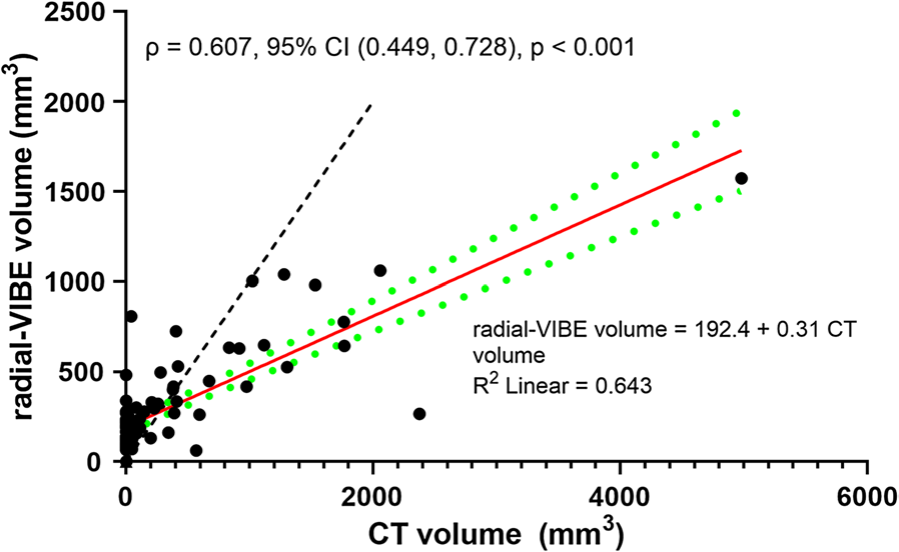
Scatterplot of thoracic aortic calcification volume measured by radial-VIBE against CT. Red solid line is the line of best fit and green dashed lines represent its 95% confidence intervals (CI); black dashed line is the line of unity. The linear regression equation and R-squared value are on the bottom right; Spearman’s rank correlation coefficient $(\rho )$, with its 95% CI and p-value, are on the top left

The median volume of calcification quantified was 191 mm^3^ (range 0–1572; IQR 189 mm^3^) by radial-VIBE and 11 mm^3^ (range 0–4982; IQR 274 mm^3^) by CT. Figure 3 illustrates the scatterplot of volume of TAC measured by radial-VIBE and CT. CT did not detect calcification in a proportion of participants (41.7%), while radial-VIBE detected signal which the algorithm reported as calcification in all of those individuals (ranging from 65 to 482 mm^3^). There was only one case when calcification was detected in CT (2.2 mm^3^) and none in radial-VIBE.

Spearman’s rank correlation coefficient was assessed as variables were not normally distributed. There was a positive monotonic correlation between radial-VIBE and CT calcification volume [$\rho$ = 0.607, 95% CI (0.449, 0.728), p < 0.001]. Linear regression equation for radial-VIBE volume was 192.4 + 0.31 CT volume.

The ICC estimates based on single-measure and 2-way mixed effects model for consistency and absolute agreement were similar [ICC for consistency: 0.537, 95% CI (0.378, 0.665); ICC for absolute agreement: 0.539, 95% CI (0.380, 0.667)].

**Fig. 4:**
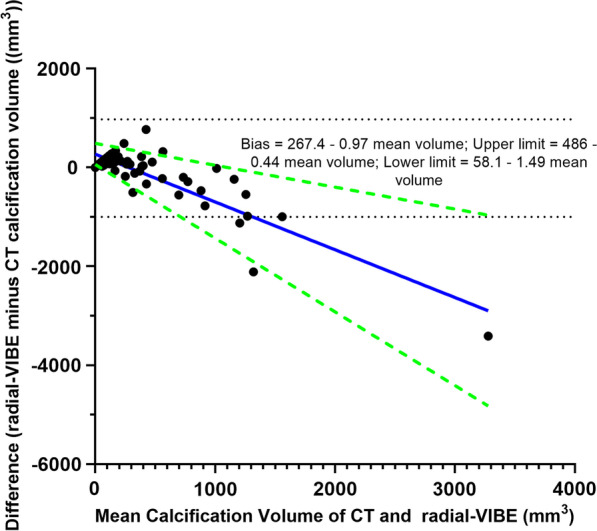
Bland–Altman plot of difference in aortic calcification volume against mean calcification volume. Difference in calcification volume = radial volumetric interpolated breath-hold examination (radial-VIBE) minus CT volume. The linear regression equations for bias and its estimated regression based 95% limits of agreement (LOA) are on the top right. Blue solid line represents bias; green-dashed lines are the estimated regression based 95% LOA; black-dashed lines are the crude 95% LOA

According to the Bland–Altman plot (Fig. 4), the bias (radial-VIBE volume minus CT volume) and standard deviations of the differences were proportional to the magnitude of mean volume (i.e., proportional bias was present). At lower calcification volume, differences tend to be positive (i.e., radial-VIBE values were higher than CT). As the mean volume of calcification detected by CT and radial-VIBE increased, radial-VIBE values decreased proportionally relative to CT values and the bias became increasingly negative.

The relationship between bias and mean calcium volume modelled with linear regression was: Bias = 267.4–1.0 mean volume. P-value of the slope (− 0.967) was < 0.001, thus confirming that the difference in volume is related to the magnitude of volume (i.e., mean volume). The 95% LOA could be visualised in Fig. 4, which illustrates that the standard deviations of the differences between radial-VIBE and CT results were also related to the mean volume.

### Intra- and inter-observer reliability

The intra-observer reliability based on absolute agreement and 2-way mixed effects model was 1.00 for CT and 0.993 [95% CI (0.957, 0.998), p < 0.001] for radial-VIBE measurements. The inter-observer reliability using the same measures was 1.00 for CT and 0.990 [95% CI (0.959, 0.997), p < 0.001] for radial-VIBE.

### Comparison with coronary artery agatston score

There was a positive association between TAC volume detected by CT and CAC Agatston score [$\rho$ = 0.404, 95% CI (0.214, 0.565), p < 0.001]. Meanwhile, there was also a positive, but weaker, association between TAC measured by radial-VIBE and CAC Agatston score [$\rho$ = 0.211, 95% CI (0.008, 0.396), p = 0.039].

### Outliers and sensitivity analyses

Three participants were identified as outliers as their standard residuals were larger than 3. The sections of aorta that were analysed on the CT and radial-VIBE in these patients were reviewed and deemed to be similar. Radial-VIBE images from 2 of the patients were of poor quality and had obvious artefact, which was likely to have influenced the results. No obvious explanation was found for the other outlier. Sensitivity analyses were performed with exclusion of these 3 outliers and the results remained relatively unchanged (results, scatter plot and Bland–Altman plot included in Additional files 2, 3).

### Qualitative assessment

**Fig. 5:**
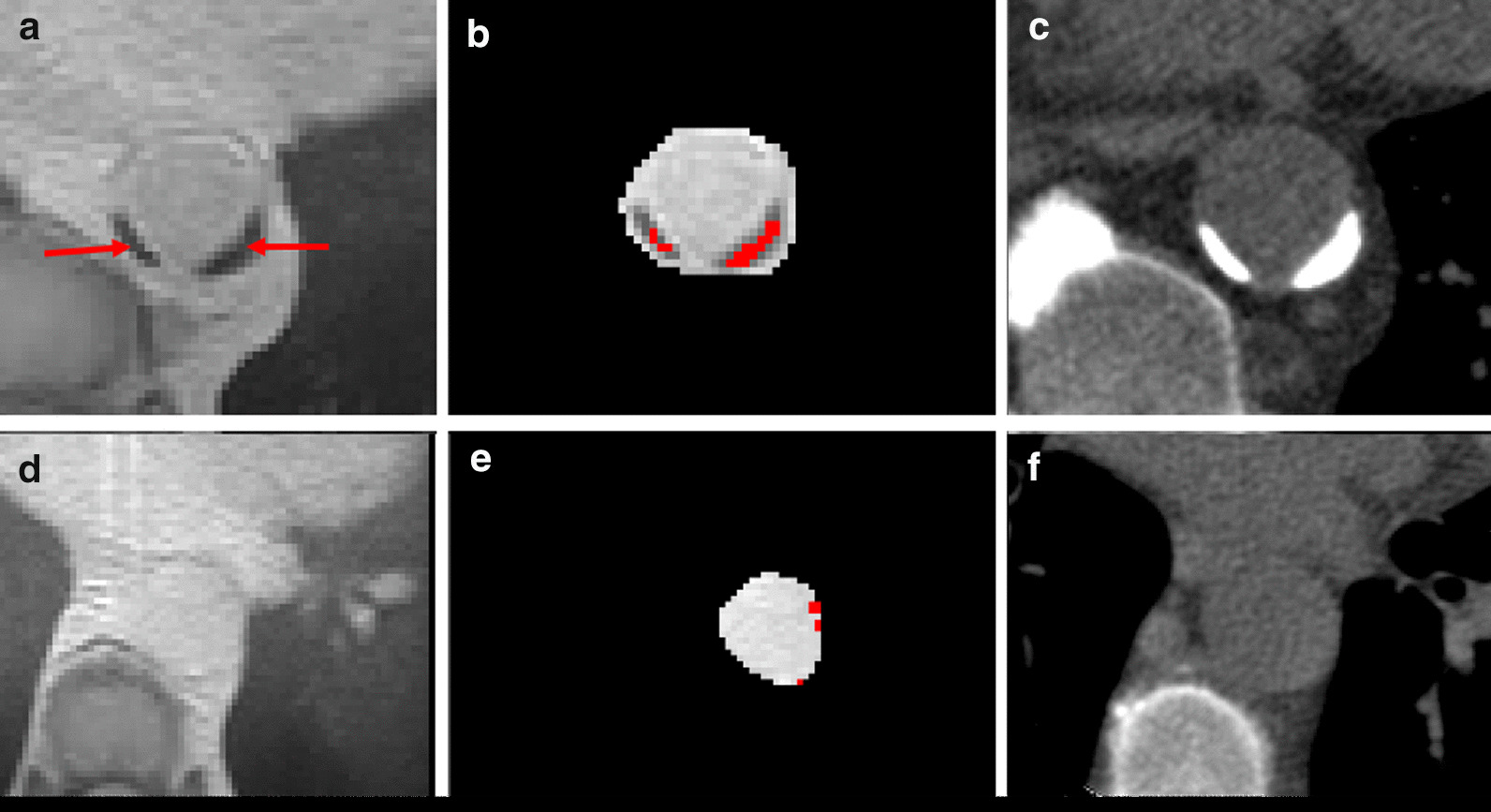
Representative images of under-detection (**a**, **b**) and over-detection (**d**, **e**) on radial-VIBE compared to CT. Images **a** and **b** are axial slices of radial-VIBE and image C is an axial slice of CT from the same patient. Red arrows on images **a** and **b** indicate calcifications, which appear as hypointense voxels. Red areas on image **b** illustrate the voxels that are considered as calcifications by the segmentation algorithm. Images **d** and **e** are identical axial slices of radial-VIBE belonging to another patient, and image F is the corresponding CT slice. Red areas on image **e** are the voxels considered as calcifications by the segmentation algorithm, which are likely to be noise and not genuine calcifications. For these two patients, the subjective analysis deemed excellent agreement between radial-VIBE and CT, despite the quantitative analysis differing significantly

The mean Likert score for the subjective, qualitative assessment of radial-VIBE compared to CT was 4.6 (SD 0.8). There was good agreement between the individual scores from the 2 observers [κ = 0.822, 95% CI (0.717, 0.927), p < 0.001]. For the subgroup of 40 participants in whom quantitative analysis showed falsely detected ‘calcification’ on radial-VIBE that was not present on CT, the mean Likert score was 4.6 (SD 0.9). A total of 31 (78%) of these scored excellent agreement for no calcification being present, while 8 were downgraded for small volume false-positive findings on radial-VIBE, with one also having a small volume of false negative. In one participant, radial-VIBE correctly identified a small area of calcification that was present on the CT, but which was subsequently removed from the CT images when the median filter was applied for quantitative analysis. Figure 5 shows case examples of when the subjective assessment of the paired scans yielded excellent agreement but the semi-automated, quantitative algorithm resulted in both over-detection and under-detection of calcification.

## Discussion

This study confirms that radial-VIBE is an option to detect and quantify TAC with a positive association when compared to the gold standard CT. On subjective assessment radial-VIBE performs well when compared to CT, but when quantifying volume using a semi-automated method, the association is imperfect and proportional bias is observed. Compared to CT, radial-VIBE over-estimates the volume of calcification when minimal calcification is present and under-estimates it when extensive calcification is present.

At lower mean volume of calcium, radial-VIBE had systematically higher volume of TAC lesions than CT. Several reasons might be responsible for this discrepancy. Firstly, it might be due to the presence of noise on radial-VIBE images, which had been falsely detected as calcification (Fig. 5). The application of a median filter to reduce noise on radial-VIBE images was attempted but it affected the detection of obvious calcium lesions as well. On the other hand, a median filter was applied on the CT images, which could have contributed to the difference. Smaller calcified lesions were possibly removed by the filter, and hence explain the over-estimation of calcification volume when low levels of calcification were present, with one definite instance of this discovered on qualitative assessment. As an exploratory post-hoc analysis, we re-analysed the 40 participants who had TAC on radial-VIBE but not on CT. Removing the CT filter for these participants did not produce a meaningful improvement in results (data not shown). It is also possible that radial-VIBE images were affected by the presence of other compounds (e.g., haemosiderin) undetected by CT, which resulted in the presence of susceptibility artefacts. Due to the use of a proton density-weighted in-phase acquisition providing a bland image contrast, the boundaries of the aortic wall were visually obscured on most radial-VIBE scans. This made accurate delineation of the aorta challenging. It was likely that non-aortic voxels were included as ROI, which could introduce more noise and affect the segmentation algorithm’s calculation of the threshold. Lastly, this study used a substantially larger voxel size than reported in a prior study of aorto-iliac and ilio-femoral calcifications [22], so that partial volume averaging of the low-signal calcifications with surrounding tissues may have been a more significant issue than in the prior study. Until improvements in radial-VIBE image quality are realised, a stepped analysis approach in which a subjective assessment is performed prior to algorithmic quantification of calcium deposits may negate some issues surrounding the false positive rate observed in the present study.

At higher mean volumes of calcium, radial-VIBE volumes were smaller than CT volumes. The reason for this could be due to blooming artefact of calcifications on CT, inappropriately magnifying dense lesions resulting in overestimation of their volume [29]. However, this assumes vascular calcification is more susceptible to blooming artefact on CT than CMR, which may not be true. Another factor could be the presence of noise within the aorta of some radial-VIBE images that could cause uneven signal intensity and inflate the standard deviation (Fig. 5). Consequently, the threshold for calcification would be extremely high and thus, diminish the segmentation algorithm’s ability to detect calcification. This was found to be the case for one of the outliers that was identified, and another one in which radial-VIBE did not pick up any calcification but the paired CT did. The same effect could also occur when very large, dense areas of calcification are present, such that the calcium signal impacts on the mean intensity of the vessel lumen and subsequently, the threshold for calcification.

This study has several strengths. Firstly, the scanning protocols were standardised to limit variability. Secondly, CT and radial-VIBE images analysed in this study were obtained within 24 h of each other, so the amount of calcification present in the patient’s thoracic aorta would be identical during the acquisition of both scans. Thirdly, by studying patients with ESRD, who have a higher vascular calcium burden than their age- and sex-matched controls [30], we could compare calcium quantification across a range of severity (as evident by the present results showing CT calcium volume ranging from 0 to 4982 mm^3^). The present findings would be applicable to other patient groups with a propensity towards vascular calcification including those with diabetes [31], CVD [30] and the elderly [32].

## Limitations

One of the limitations of this project was the manual delineation of aorta in the analysis of radial-VIBE images, whereas CT images were analysed with a more automated approach. Besides the poor definition of aortic wall in radial-VIBE images impairing the accuracy of manual segmentation as previously mentioned, manually drawing ROI was time-consuming and was less reproducible than CT analysis. However, several reasons led to that decision. Unlike CT, where the Hounsfield unit is calibrated with reference to water and its values are comparable among patients, the absolute signal intensity in CMR is not. The signal intensity is affected by various factors, which include proton density, pulse sequence, types and strength of magnet used for scanning [33]. Thus, the magnitude of signal intensity of the same tissue (e.g., heart) may vary across individuals. Consequently, it meant that calcium has no ‘absolute’ value which we could use for thresholding and segmentation. Instead, thresholding calcification in CMR images was dependent on its relative signal intensity compared to surrounding voxels—hence we used signal intensity greater than 2.5 SD below the mean signal intensity of ROI. This approach for thresholding calcifications in CMR images was used by Serhal et al. in their study, where 2 and 3 SD below the mean were classified as calcifications in the aorto-iliac and femoral arteries, respectively [22]. Due to the poor image quality of some scans in this study (often due to loss of signal from the large field of view/participant body habitus), 2 SD was not used as it could not sufficiently distinguish calcifications from noise, while 3 SD would significantly impair the detection of calcium with relatively low signal intensity. Furthermore, the thoracic aorta is near the air-filled lungs which are also hypointense. Segmenting the aorta while avoiding the lungs was crucial in order to prevent the mistake of identifying voxels in the lungs as calcification. Initially, the creation of a mask of the aorta from HASTE (Half-Fourier-Acquired Single-shot Turbo spin-Echo) images to segment the aorta was explored. Unlike radial-VIBE images, HASTE images have good aortic definition and manual segmentation is easier. Unfortunately, it was not possible to use the HASTE mask to subtract surrounding tissues from the radial-VIBE images due to different image parameters on the acquired scans (e.g., slice thickness and resolution).

Another limitation was that the typical CT scans for CAC do not cover the aortic arch and proximal descending thoracic aorta, which made it more challenging to find a common anatomical landmark in CT and radial-VIBE images to ensure that similar segments of the aorta were analysed. Additionally, the aortic arch and proximal descending thoracic aorta have been shown to be the areas of the aorta most prone to calcification [34] and it would have been useful to compare the detection of calcification by radial-VIBE and CT in those aortic segments.

The discrepancy in TAC volume between radial-VIBE and CT suggests that CT should remain the primary modality for assessing vascular calcification in clinical practice. Furthermore, CT has the advantages of wider availability, lower cost, and faster acquisition time compared to radial-VIBE. However, there is no doubt that radial-VIBE can detect vascular calcification and, if improvements in image quality are realised, it may be a plausible alternative to CT in the future, particularly where patients are undergoing CMR angiography studies where additional information on calcified plaque can be valuable. The near-future role of radial-VIBE is perhaps best suited to realm of research, where its lack of ionising radiation can allow serial imaging, either as part of longitudinal study trying to monitor the progression of vascular calcification, or to assess the impact of therapeutics in clinical trials. Radial-VIBE has the additional benefit of allowing other CMR sequences to be acquired, thus providing information on the possible effects of therapies on cardiac structure and function, as well as aortic distensibility. However, ensuring adequate image quality would be essential to allow accurate quantification of TAC using radial-VIBE sequence.

## Conclusions

This study supports the hypothesis that radial-VIBE can detect TAC. However, there is proportional bias in the measurement of calcium volume by radial-VIBE compared to CT. Quantification of vascular calcification by radial-VIBE remains a promising area for future research, but improvements in image quality (e.g., through the use of optimised protocols, smaller voxels and motion correction) are necessary.

## Supplementary Information

**Additional file 1.** Flowchart of cardiovascular magnetic resonance (CMR) and computed tomography (CT) scans available for analysis.

**Additional file 2.** Sensitivity Analysis (with 3 outliers excluded) Results and Scatterplot.

**Additional file 3.** Sensitivity Analysis Bland-Altman plot.

## Data Availability

Source data will be provided upon reasonable request.

## Abbreviations

CAC: Coronary artery calcification
CI: Confidence interval
CKD: Chronic kidney disease
CMR: Cardiovascular magnetic resonance
CT: Computed tomography
CVD: Cardiovascular disease
eGFR: Estimated glomerular filtration rate
ESRD: End-stage renal disease
FOSS: Free and open source code software
FOV: Field-of-view
HASTE: Half-Fourier-Acquired Single-shot Turbo spin-Echo
HU: Hounsfield units
ICC: Intraclass correlation coefficient
IQR: Interquartile range
LOA: Limits of agreement
Radial-VIBE: Radial volumetric-interpolated breath-hold examination
ROI: Region-of-interest
SD: Standard deviation
TAC: Thoracic aortic calcification
TE: Echo time
TICKER: The Interrogation of the Cardiomyopathy of Chronic Kidney Disease With advancEd caRdiac Imaging
TR: Repetition time
ViKTORIES: Vitamin K in kidney transplant organ recipients: investigating vEssel stiffness
