# Supplementary material for: Cardiovascular magnetic resonance for the detection of descending thoracic aorta calcification in patients with end-stage renal disease
Source: J Cardiovasc Magn Reson. 2021 Jun 24;23:85. doi: 10.1186/s12968-021-00769-6 (PMC8223384; doi:10.1186/s12968-021-00769-6)
Supplement: Supplementary file 1 — Additional file 1. Flowchart of cardiovascular magnetic resonance (CMR) and computed tomography (CT) scans available for analysis. [file 12968_2021_769_MOESM1_ESM.pptx]

## Slide 1
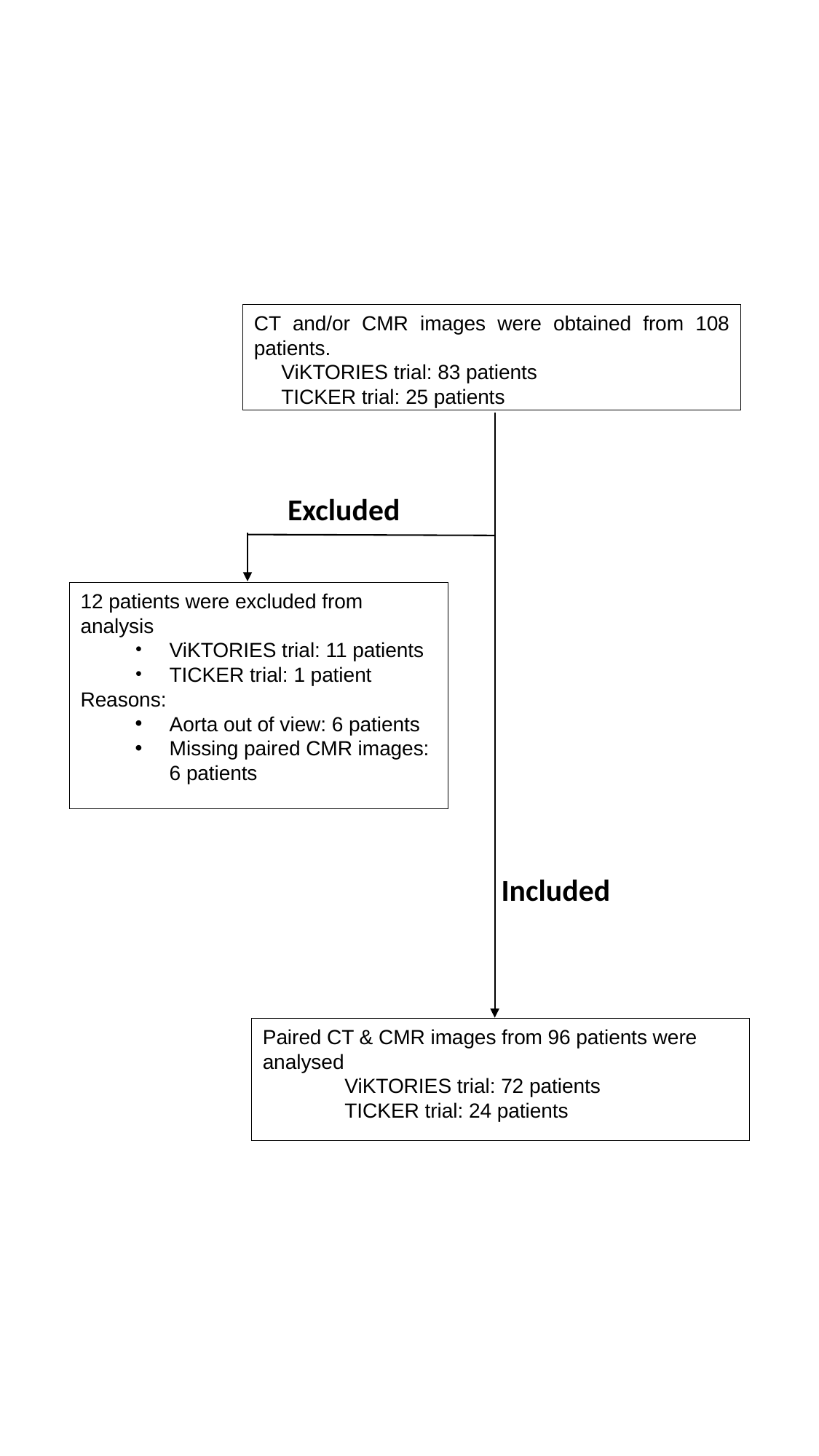

CT and/or CMR images were obtained from 108 patients.
ViKTORIES trial: 83 patients
TICKER trial: 25 patients
Excluded
12 patients were excluded from analysis
ViKTORIES trial: 11 patients
TICKER trial: 1 patient
Reasons:
Aorta out of view: 6 patients
Missing paired CMR images: 6 patients
Included
Paired CT & CMR images from 96 patients were analysed
ViKTORIES trial: 72 patients
TICKER trial: 24 patients
