# Supplementary figures and images for: Cardiovascular magnetic resonance for the detection of descending thoracic aorta calcification in patients with end-stage renal disease
Source: J Cardiovasc Magn Reson. 2021 Jun 24;23:85. doi: 10.1186/s12968-021-00769-6 (PMC8223384; doi:10.1186/s12968-021-00769-6)

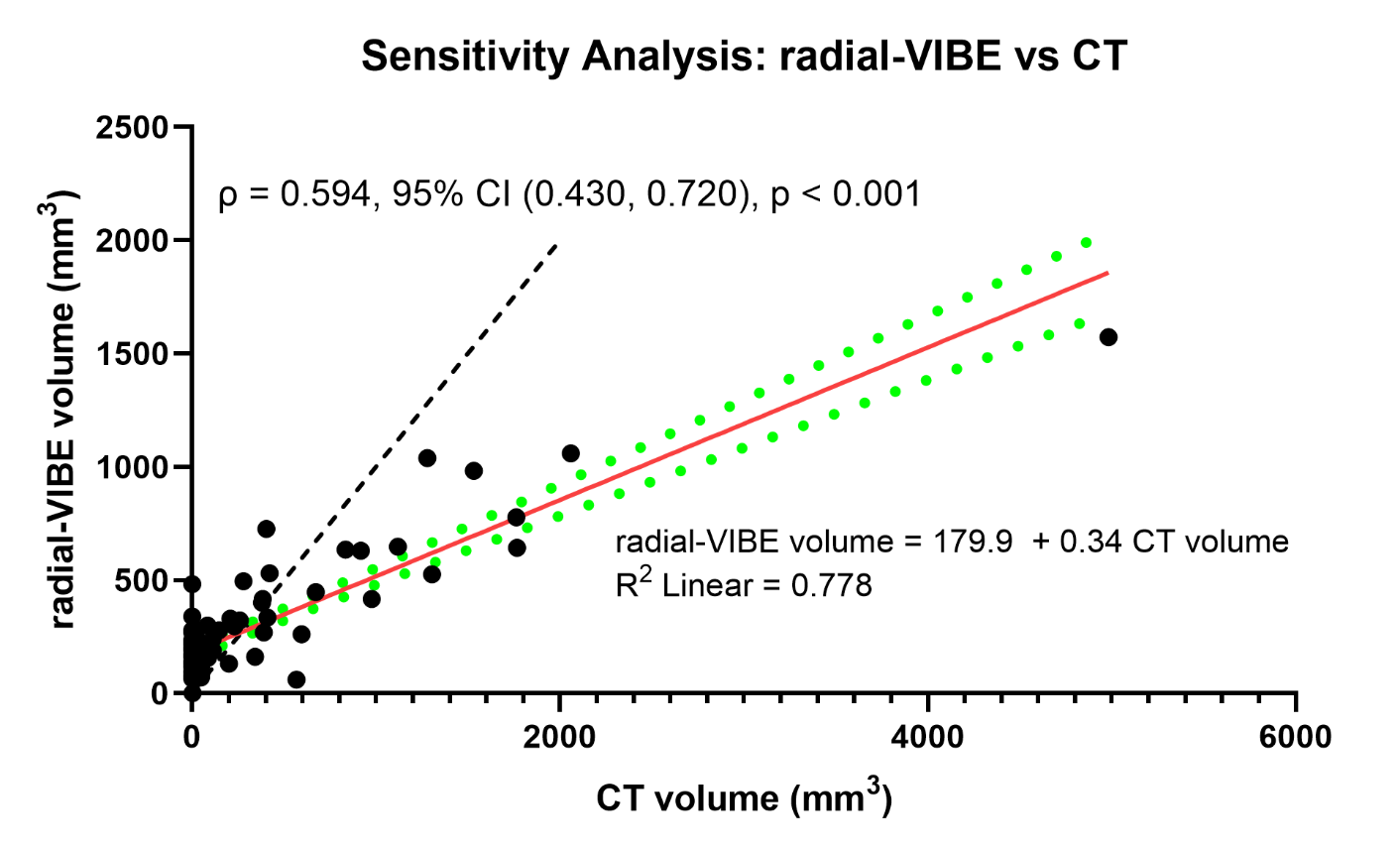


Additional file 2 Sensitivity Analysis (with 3 outliers excluded) Results and Scatterplot

Supplement: Supplementary file 2 — Additional file 2. Sensitivity Analysis (with 3 outliers excluded) Results and Scatterplot. [file 12968_2021_769_MOESM2_ESM.docx]

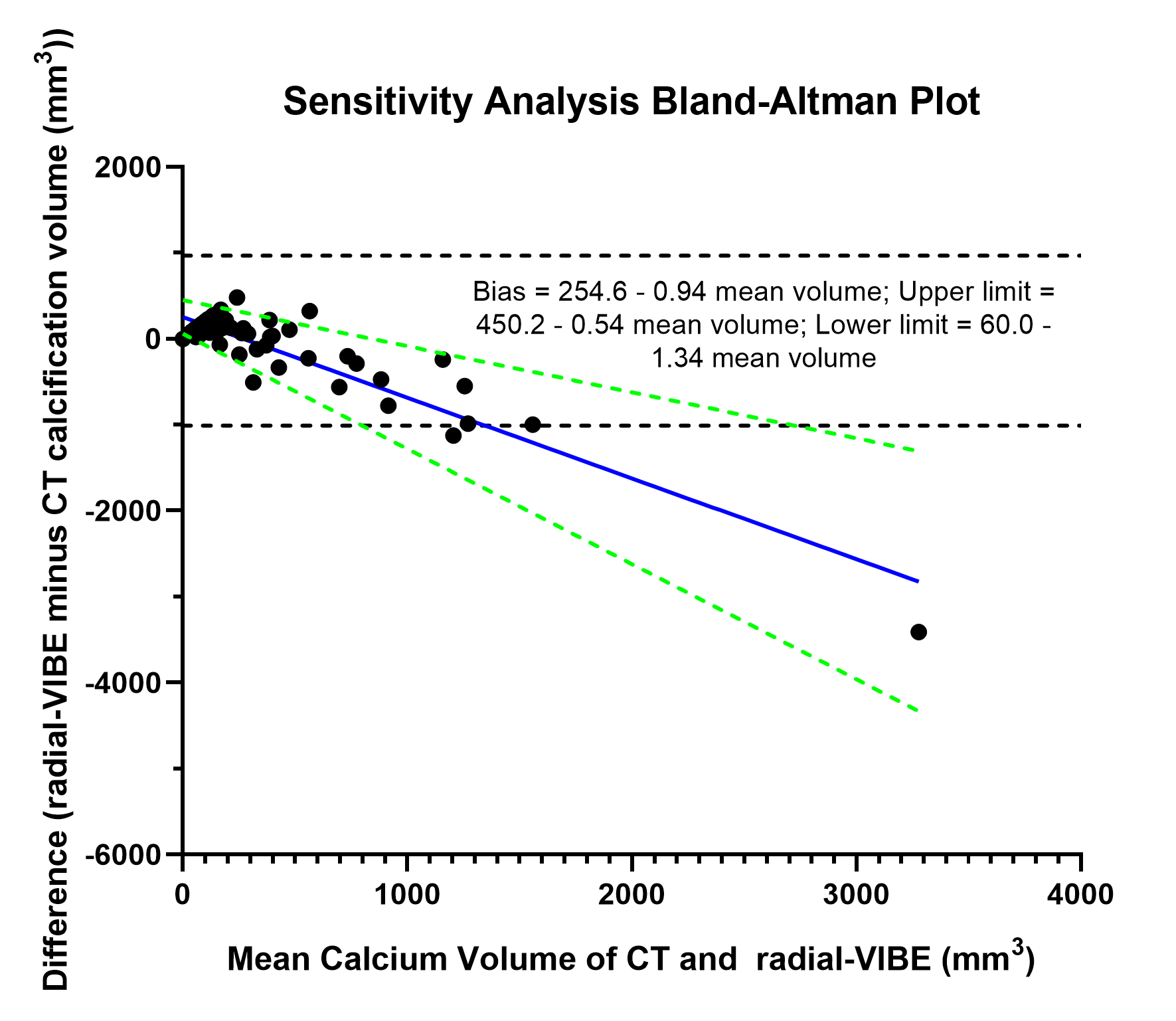


Additional file 3 Sensitivity analysis Bland-Altman plot

Supplement: Supplementary file 3 — Additional file 3. Sensitivity Analysis Bland-Altman plot. [file 12968_2021_769_MOESM3_ESM.docx]
